# Health Transparency and Communication on the Government Websites of Ibero-American Countries: The Cases of Chile, Colombia, Ecuador, and Spain

**DOI:** 10.3390/ijerph18126222

**Published:** 2021-06-08

**Authors:** Daniel Barredo Ibáñez, Pedro Molina Rodríguez-Navas, Narcisa Jessenia Medranda Morales, Vanessa Rodríguez Breijo

**Affiliations:** 1 School of Human Sciences, Universidad del Rosario, Cr. 6 No. 12C-13 Of. 517, Bogotá 111821, Colombia; 2 Fudan Development Institute, Fudan University, Shanghai 200433, China; 3 Department of Audiovisual Communication and Advertising, Universitat Autònoma de Barcelona, Edifici I, Despatx 308, Campus de la UAB, Bellaterra, 08193 Cerdanyola del Vallès, Barcelona, Spain; pedro.molina@uab.cat; 4 Social Communication Career, Universidad Politécnica Salesiana, Av. Isabel la Católica N23-52 y Madrid, 170517 Cuenca, Ecuador; nmedranda@ups.edu.ec; 5 Department of Communication Sciences and Social Work, Universidad de La Laguna, Camino La Hornera, 37, 38205 San Cristóbal de La Laguna, Santa Cruz de Tenerife, Spain; vrbreijo@ull.edu.es

**Keywords:** health communication, digital communication, transparency, Ibero-America, institutional communication

## Abstract

Through the strategic use of health communication from their websites, government institutions can achieve greater promotion and prevent health issues for citizens, at whom such websites are aimed. Thus, the transparency of these sites is essential to favor issues such as participation, accountability, and good governance. In the present study, through content analysis, we examined how active transparency and communication in health is built through analyzing the information provided by the different administrative levels with competencies in this field (government, regions, and municipalities) from the following countries: Chile, Colombia, Ecuador, and Spain. The results were projected according to a double axis of analysis. On the one hand, we offer a description of the levels of management of this phenomenon (structural characteristics and available resources). On the other hand, we developed indices based on each of the countries to compare the typologies grouped in these Ibero-American countries. As a general conclusion, the results evinced insufficient transparency in common; however, the deficit was less in countries that had a public health system.

## 1. Introduction

Public administration transparency implies that the actions carried out by institutions are known and reviewed [1]. Through transparency, information on actions and forecasts is provided, thereby accounting for every cent spent in the framework of public and political activity and detailing all income and expenses, including the real salaries of politicians, officials, and the workforce [2]. Therefore, it is considerably common to find a strong connection between the concept of transparency in public administration and the prevention of corruption because the incidence of illegal acts is expected to be less likely, given the knowledge of and monitoring by public management, than it would be in a situation of secrecy and opacity. From this perspective, citizens might be conceived of as auditors [3]. This function is understood as a right because citizens provide the money that the administration manages through taxes, thereby making it pertinent for them to know how the said money is being used [4]. Thus, granted access to information concerning government management, citizens can evaluate such management and ensure that politicians who have not carried out adequate administration do not regain power [5]. Therefore, transparency is both a preventive mechanism for corruption and an instrument for accountability. Furthermore, the availability of public information encourages political participation by citizens [6] as well as the dissemination of institutional information as sources for the media [7].

However, in operational terms, the scope that transparency should have remains a contentious matter. Some authors demand a total openness of information, using terms such as “radical transparency” [8] or “hypertransparency” [9], or for the options available on websites to be personalized [10] by proposing participation methods to agree on evaluation methodologies [11] or civic audits [12], whereas others question how transparency has been addressed in such studies [13].

These differences have motivated some research that compares transparency among countries and regions of the world to determine the influence of different communicative and political cultures. Bolívar et al. [14], for instance, evaluated the use of the web to disseminate government information in three areas—namely, Anglo-Saxon, South American, and continental European regions—and verified the influence of the different administrative traditions. Grimmelikhuijsen et al. [15] performed a comparative analysis between the Netherlands and South Korea and concluded that transparency is more valued in countries with a lower tradition of access to information. Huijboom and van den Broek [16] contrasted the open data strategy in five countries—Australia, Denmark, Spain, the United Kingdom, and the United States—and identified important differences in how information was published. Finally, Gallego-Álvarez et al. [17] established a correlation between municipalities that had implemented a high level of technology with the development of e-government. Other works have also addressed national differences by observing the behavior of local entities [18,19,20]. These results indicate the importance of institutional and other economic factors that can hinder the implementation of digital strategies. In addition, in such national comparisons, the importance of analytical bias deriving from applying methodologies designed on the basis of the political sciences or from a communicative perspective has been observed [21].

This comparative perspective is the one that we have adopted in the present research study, which aims to analyze the content of the government websites of four Ibero-American countries—Colombia, Chile, Ecuador, and Spain—at different administrative levels to ascertain whether these websites offer sufficient information that is of high quality in the specific field of health. These countries have been chosen because despite the differences in their political–administrative systems and legislations, they represent two big trends of health organizations: administrative contexts within mixed public–private health systems (Chile and Colombia) and those within public health systems (Ecuador and Spain).

## 2. Transparency in Chile, Colombia, Ecuador, and Spain

Transparency is described as information access in order for the citizens to find out, if necessary, both the planning and the administration of public resources [2]. As it is explained in this epigraph, the four countries chosen have approved laws that regulate transparency. However, one essential difference that we can advance is linked both to state control and transparency cultures [14]. Hence, from this angle, transparency depends on the law, and above all, it depends on the way states monitor its compliance, the values that have been adopted in public administration, and the level of citizen participation [5,6].

In that sense, Chile is considered the leading Latin American country in terms of the development of public administration [22]. In 2008, Law No. 20,285 on the Access to Public Information [23] was passed, considering it a right, establishing active and passive transparency procedures, and creating the Council for Transparency (CPLT, in Spanish), a body that is capable of providing sanctions [24].

However, eight years later, Pefaur and Moreno [25] found a confidence crisis in the country’s institutions because of the lack of transparency and the prevalence of corruption. These authors concluded that the modernization of the country did not require new legislation but rather successful implementation of the existing legislation. To that end, the aforementioned authors employed indices that were prepared by the Council for Transparency of Chile (at https://www.consejotransparencia.cl, accessed on 28 April 2021), of which they are a part. The indices confirmed that very few citizens were aware of their right to access information. The National Transparency Study of 2019 [26] continued to show this distrust toward, and distancing from, institutions, as well as the perception that access to public information is difficult. Similarly, Hernández [24] observed a “normative rigidity” that, despite making it easier for administrations to comply with the Law, did not lead to better local governance. Abeleida et al. [27] revealed one of the key aspects of this problem by noting that the access and use of documents remains complicated even when complying with legal mandates.

In the Colombian case, transparency is regulated by Law No. 1,712 of 2014: Article 24 establishes the Right of Access to Information, of a universal nature, which can be claimed by anyone when the information is public through Article 25. The only exception established by this regulatory framework is the so-called “reserve of national security and defense or international relations” (Art. 27), in which case an administrative court will be the one to resolve a citizen’s request.

However, as is the case in Chile, effective implementation of transparency is far from becoming a reality despite the normative and institutional foundation that supports it. It is made difficult by a culture of corruption, weak institutionalization in some of the territories, and the low participation of the community because of the armed conflict that, for decades, has devastated the country [28]. Católico and Santos [29] studied Territorial Public Administrations in 17 departments related to the consolidation of peace and showed that the implementation of laws and procedures was encouraged less in addition to there being low citizen participation in those contexts wherein there was a greater prevalence of corruption and vice versa. Ortiz and Beltrán [30] evaluated the transparency of Colombian municipalities and found a link between this and the efficiency of municipalities concerning spending, investments in infrastructure, and human contracting.

In Ecuador, a substantial legislative effort has been made in this area. The Constitution, which establishes the Function of Transparency and Social Control, the implementation of the Council for Citizen Participation and Social Control, the Organic Law of Transparency and Access to Public Information (LOTAIP, in Spanish) of 2004, and the Organic Law of Citizen Participation of 2010, is clear evidence of the same [31].

The drawback of the LOTAIP is that it responds to the 1998 Constitution and not the current one of 2008. Its benefit is that it guarantees access to information. Municipalities, for example, must publish their documents on their respective web portals, according to Article 7 [32]. The Law applies to all institutions that are part of the state and institutions that receive state funds. However, despite this legislative effort, the particular interests and arbitrariness of political leaders [33] limit democratic access to information.

There is a distance between the elite, who assume the concepts of transparency and public information, and the intermediate public officials, who do not have the necessary political will. Furthermore, the Ecuadorian state has not reached the final stage of institutional maturity that transparency requires. Moreover, officials and workers do not have the necessary training in matters of transparency. These three factors make the implementation of the legislative framework and the necessary instrumentalizations the cause for Ecuador not having achieved the expected level of efficiency [31].

As for Spain, if transparency is defined as the duty of public powers to account for their actions, the first legislation that regulated it in the European Union was Regulation (EC) No. 1049/2001 of the European Parliament and of the Board of 30 May 2001 [34]. In Spain, Law No. 19 of 2013, on transparency, access to public information, and good governance, was the result of the adhesion to the Open Government Alliance and the Council’s Alliance for Open Government of Europe. It responded to the commitment to bring citizens closer to the administration, to be accountable, and to encourage participation [35]. In terms of the promulgation of this law, almost all autonomous communities in the country have created their own versions of this by implementing the national one.

Furthermore, transparency in Spain has been evaluated to ascertain the degree of compliance with the Law. Some research has addressed public communication by Spanish city councils through transparency indicators and information quality, georeferencing the results in the *Mapa Infoparticipa* [36]. The evolution of public Spanish portals regarding the availability and democratic access of information by citizens has seen its fair share of research [37]. The degree of compliance by Spanish city councils with the 20 minimum data sources published, as agreed in the Strategic Guide of the Spanish Federation of Municipalities and Provinces, has been a research topic [38]. The methodology of analyzing open data portals of municipalities has been studied [39]. The quality of the disclosure of data related to transparency and accountability in the autonomous Spanish communities for 2013–2017 has been a topic of research interest [40]. The organization of the information in the Portal of Transparency of Spain and the availability of the information and of the subjects bound by the Law of transparency, access to public information, and good governance have been researched [36]. The classification of the open data portals of the regional governments of Spain within the models of “sincere transparency” or “apparent transparency” has had minds thinking [40]. Finally, the differences in the data obtained in 2013, 2015, and 2018 have been put under the scanner as well [41]. All of the aforementioned studies, whether to a greater or lesser extent, concluded that there is still a long way to go in the implementation of the regulations on access to public data.

## 3. Health Systems of Chile, Colombia, Ecuador, and Spain

Chile’s 1980 Constitution configured a health system wherein the great importance of private versus public management is glimpsed. Article 19, no. 9 establishes that the state must protect “the free and equal access to actions of promotion, protection, and recovery of the health and rehabilitation of individuals” as well as exercise the “coordination and control of health-related actions.” In the same point, it is said that “each person will have the right to choose the health system they wish to benefit from, whether state or private”; thus, the state must “guarantee the execution of health actions, whether they are provided through public or private institutions, in the form and conditions determined by law, which may establish mandatory contributions.”

In 2014, a Presidential Advisory Commission concluded that this system, which is based on competition and freedom of choice, should be reoriented to the right to health and principles of solidarity and equity [42]. However, subsequent studies continue to confirm the persisting segregation of the population, and criticisms have noted that health has been understood as a consumption good [43,44].

Similarly, in Colombia, the 1991 Constitution established “physical integrity” as one of the fundamental rights (Article 44) while entrusting the provision of health services to the state (Articles 49 and 366). Law No. 100 of 1993, of December 23, created the Comprehensive Social Security System, with which the state guarantees all citizens’ access to the said system (Article 3), establishes its supervision to the state organism, and establishes its provision to public or private institutions (Article 4). However, the health system has been undergoing gradual reforms to decentralize the provision of services. Thus, with Law No. 1122 of 2007, provisions were established to regulate requests from Health Service Provider Institutions (IPS, in Spanish) and Health Promoting Entities (EPS, in Spanish). Despite being largely dominated by private companies, Colombia has a National Health Superintendency that is in charge of ensuring matters in case of divergences between EPS and users. Later, Law No. 1438 of 2011 established the guiding principles of the system, among which transparency (Article 3.14) stands out and was proposed on the basis of provider mechanisms, policies, and the link between operators. Through the constitutional articles of 1991, and with the indicated normative context, the General System of Social Security in Health of Colombia was developed in 1993 and is of a mixed public–private type, with a contributory base of contributors and financing resources fed through taxes to the entire nation [45]. Suárez et al. [46] confirmed, on the one hand, the dissatisfaction of users when they came across irregular qualities of the health system, and on the other hand, the lack of financial stability of the EPS.

Likewise, the Political Constitution of Ecuador (2018) established in Article 12 that health is a right that the state guarantees. In addition, this article indicates that the provision of health services is governed by the principles of equity, universality, solidarity, interculturality, quality, efficiency, efficacy, precaution, and bioethics with a gender- and generation-based approach. The Organic Health Law (2006) indicated that the highest health authority in Ecuador is the Ministry of Public Health, which is responsible for applying, controlling, and monitoring compliance with the Law.

Lucio et al. [47] indicated that the National Health System of Ecuador involves both public as well as private sectors. The first serves 51% of the population and includes the Ecuadorian Institute of Social Security, the Institute of Social Security of the Armed Forces (ISSFA, in Spanish), the Institute of Social Security of the National Police (ISSPOL, in Spanish), the programs of the Ministry of Economic and Social Inclusion (MIES, in Spanish), and the corresponding municipality-level institutions. The private sector serves 49% of the population with private doctors or clinics. This sector is becoming increasingly important, given the difficulties faced by the public sector in providing service [48]. There is also a private nonprofit sector, with independent civil society and social service organizations [49].

Finally, the National Health System in Spain has universal coverage and is financed with public money from taxes and the copayment of medicines and health products by patients. This copayment can be reduced or eliminated depending on the group to which the patient belongs or the nature of their treatment. There are also insurance and private health centers; however, unlike in Chile and Colombia, care in Spain is provided mainly through the public network. The 17 autonomous communities of the country are in charge of providing medical care and promoting health. The central government is in charge of formulating legislation and coordinating cooperation between the different institutions and agents. It does so through the Ministry of Health, Consumption, and Social Welfare [50].

Regarding the local level, Law No. 14 of April 25, 1986, General Health, establishes that “[t]he regulations of the Autonomous Communities, when providing for the organization of their respective health services, must take into account the responsibilities and competencies of the provinces, municipalities, and other intra-community Territorial Administrations, in accordance with the provisions of the Statutes of Autonomy, the Local Government Law, and this Law.” Therefore, the competencies of local areas are defined in each regional health regulation.

## 4. Materials and Methods

### 4.1. Research Design

This study, of a non-experimental nature, has a descriptive and correlational scope and was designed on the basis of the *Infoparticipa* project [12], which presented a methodological proposal with which to evaluate the transparency of Spanish local public administrations on their websites from a communicative perspective. This means that the importance of publishing complete, as well as understandable, accessible, and reusable, information is considered to enable citizens to monitor and evaluate the actions of elected political leaders and facilitate their participation in public affairs.

In the *Infoparticipa* project, a list of 52 indicators is proposed to examine transparency in public administration [12]. In this study, we have created a new version to address a new field, that of health, from a comparative international perspective, considering that each country has a different political and administrative organization, as well as its own legislation. To achieve this, we made the decision to reduce the list of indicators to a few basic questions so that data in all national cases at three administrative levels (state, region, and municipality) could be obtained, assuming that the different levels in each country have different competencies but that citizens, as a whole, must be able to know the fundamental data on health management in their country. To carry out the reduction process, only the aspects common to the different scales of the administration have been considered, as well as those comparable with respect to the characteristics of each national administration. Thus, indicators on the information that identify members of the opposition in municipal councils have not been considered because in Colombia, for example, the mayor is elected by direct suffrage; hence, comparison with the Spanish case, where the councilors are elected in the municipal elections, is not possible, given the different nature of their position. Similarly, for this same case, in national administrations, it is not pertinent to consider that the opposition be reported on ministerial websites because political groups that are not part of the government exercise their work of control of the executive in the national parliament and not in the ministerial institution. Therefore, indicators on bodies that are exclusively municipal, such as plenary sessions or government boards, have not been considered because this would have implied making an analysis with different indicators for each level of administration and for each country. On the contrary, in this work, we have chosen to carry out an analysis common to the four countries, which allows us to compare the level of information on health administration that citizens receive.

Subsequently, the procedure has been adapted to analyze other levels of administration in Spain, such as the provincial councils or the regional councils of Catalonia. In addition, other adaptations have been made to implement it to the local administrations of Ecuador [32] and Colombia [51] as well as to other types of entities, such as the field of mining [52], or the national television of Ecuador [53]. For each case, the characteristics of the public administrations or entities investigated have been studied as have the national legislations on transparency.

Once the list of indicators had been defined, considering the legislation and administrative characteristics of each country, a pre-test was carried out. Furthermore, after these double validation processes were performed (theoretical and empirical), we obtained a list of 24 indicators that assess whether information is offered in all areas where fundamental questions are proposed therein. This includes information on the people responsible for government and management, that is, political leaders and senior officials (indicators 1–6); resource management (indicators 7–19); and communication and facilitation of participation (indicators 20–24).

### 4.2. Measurements

The analysis sheet that was used to operate the content analysis contained 24 indicators, which were measured through dichotomous responses (0 = no; 1 = yes). The indicators were evaluated positively when the information was found on the analyzed public administration website but not when there was only a link that led to another organization’s website, even if this was a national transparency portal. Questions about the comprehensibility, ease of access, and other aspects related to the quality and timeliness of the information have not been considered because this analysis makes an approximation of the questions, which also evinces the difficulties of a comparative analysis between different political–administrative contexts despite being an issue of international relevance.

The measurement given to each indicator regarding the total count was always constant because the *Infoparticipa* procedure considers that all the questions raised are important from a communication perspective in that their objective is not only to reveal or act as a barrier against corrupt practices but also to promote citizen participation and accountability.

Coding was carried out by four experts who were a part of the study during the months of March and April 2021. To identify the reliability of the sheet, once data collection was completed, the Kuder–Richardson coefficient (KR20), which is often used to test the cohesion level of dichotomous variables, was used. The KR20 coefficient yielded a value of 0.831 for the 24 elements; this coefficient is considered highly acceptable because it approaches 1 [54]. In relation to data interpretation techniques, along with an analysis of frequencies, a chi-square test was conducted to examine the association between variables, along with Cramer’s V index, which helps typify the intensity of the said association. The data were analyzed using SPSS Version 25 software.

### 4.3. Sample

In total, 64 case studies were selected, with 16 studies for each of the four countries. The case studies were selected through nonprobabilistic sampling that used quotas; the selection was based on the following characteristics of pertinence and relevance.

- The website of the Ministry of Health of each of the four countries has been included: the Ministry of Health (Chile); the Ministry of Health and Social Protection (Colombia); the Ministry of Health (Ecuador); and the Ministry of Health, Consumption, and Social Welfare (Spain).
- The websites of the health areas of the five autonomous communities, prefectures, or departments with the largest populations in these contexts have been included: the regions of Metropolitana, Valparaíso, Biobío, Maule, and La Araucania (Chile; the website of the Regional Health Secretariat or SEREMI in each region was reviewed); the departments of Antioquia, Cundinamarca, Valle del Cauca, Atlántico, and Santander (Colombia); the prefectures of Guayas, Pichincha, Azuay, Manabí, and Cotopaxi (Ecuador); and the autonomous communities of Andalusia, Catalonia, Madrid, Valencian Community, and Galicia (Spain).
- The websites of the health areas of the 10 most populated municipalities in each country have been included: Puente Alto, Maipú, Santiago, La Florida, Antofagasta, Viña del Mar, San Bernardo, Valparaíso, Las Condes, and Temuco (Chile); Bogotá, Medellín, Cali, Barranquilla, Cartagena, Cúcuta, Soacha, Soledad, Bucaramanga, and Bello (Colombia); Guayaquil, Quito, Cuenca, Machala, Ambato, Esmeraldas, Riobamba, Portoviejo, Ibarra, and Naranjal (Ecuador); and Madrid, Barcelona, Valencia, Seville, Zaragoza, Malaga, Las Palmas de Gran Canaria; Murcia; Bilbao, and Palma de Mallorca (Spain).

## 5. Results

### 5.1. Description of the Management Levels

In Table 1, we present both frequencies and implementation percentages for each indicator and country examined. The results are described according to the ministries (*M*), the departments, prefecture, or autonomous communities (*R*), or the municipalities (*Mu*). At the same time, in the column “total,” we have added all the data together to facilitate comparison between the indicators. In the items examined, the highest scores, globally, were obtained because of the existence of a button or section, within each website, called “Transparency” or something similar (54/64 = 84%), which reached total, or very high, compliance in all cases except on the websites of the regions of Chile (2/5 = 40%) and Colombia (2/5 = 40%). They could also be attributed to the publication of the annual budget (54/64 = 84%), implemented in all cases, with the exception of the departments of Chile (2/5 = 40%), with a moderate level. The availability of information on budget execution and annual statements (52/64 = 81%), which was only not found in the Ministries of Chile and Spain, and which reached a moderate level in the Chilean departments (2/5 = 40%) despite being one of the most important indicators of transparency in public administration, was another factor [2]. In addition, the availability of information on annual and multiyear plans and programs (51/64 = 80%), with a lack of implementation in the Ministry of Ecuador, and with a moderate presentation in the municipalities of Chile (5/10 = 50%) and Spain (5/10 = 50%), could be deemed factors.

Following the four referenced indicators, which were present in approximately eight of the 10 websites in the health area examined, we found a greater difference with respect to the other items, with a distance of 10 or more percentage points.

Among the less frequent items, we found the publication of the institutional agenda of the health minister of the ministry, territory, or municipality (8/64 = 13%), which is nonexistent in all cases addressed in Chile and Colombia, as well as the Ministries of Ecuador and Spain. Similarly, the publication of the characteristics of institutional advertising campaigns in the media registered a low overall frequency (12/64 = 19%), nuanced by their inclusion in the Ministries of Chile and Spain, as well as with the total compliance of the Spanish Autonomous Communities.

The web pages studied tended to restrict the incorporation of some type of strategy to favor the accessibility of ethnic groups or nationalities (17/64 = 27%) despite total compliance of the Ministry of Spain and the high levels of compliance in the territories of Chile, Colombia, and Spain (3/5 = 60%). Similarly, the institutions that constituted part of the sample exhibited resistance to publishing the resumes of senior officials related to the area (18/64 = 28%) except in the cases of the Ministries of Chile and Colombia, as well as in the autonomous communities (4/5 = 80%) and the municipalities (7/10 = 70%) of Spain.

**Figure 1:**
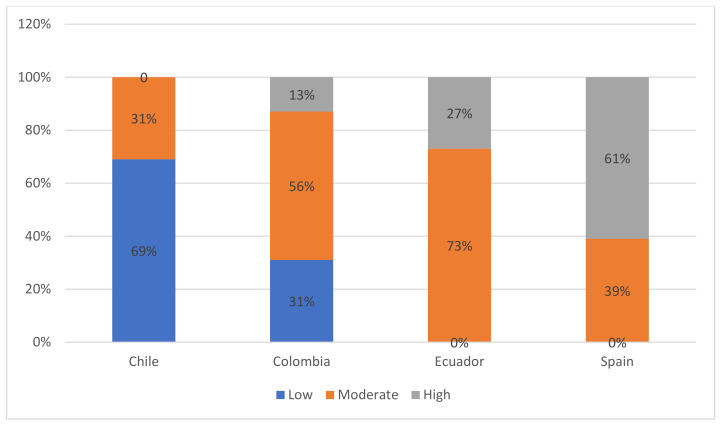
Levels of transparency grouped in the websites of the health areas of the ministries, regions, and municipalities of Chile, Colombia, Ecuador, and Spain (2021). Source: authors’ elaboration.

After disregarding four missing values, we found an association with a strong intensity between type of country and level of transparency observed (χ^2^ (6, N = 60) = 34,190, Cramer’s V = 0.755, *p* < 0.05). This association depends on some aspects surrounding the evaluation carried out, such as the regulatory frameworks approved and the state control of the countries examined. To understand the data presented in Table 1 better, we elaborated on an index comprising the 24 variables of the research study through three ranges calculated on the basis of the total range of 19 data points recorded in the said summation. Hence, in Figure 1, we have added all the cases together for each country analyzed, and then, we have created a ranking with three categories (low; moderate; high), calculated statistically. Thus, a high level of implementation of indicators was achieved in Spain, with a value of 61% overall, when compared with 27% in Ecuador, 13% in Colombia, and 0% in Chile, within the range that accounted for the highest frequencies of compliance.

On the other side of the spectrum, Chile (69%) and Colombia (31%) were awarded the lowest levels of compliance with the proposed indicators of transparency on the websites of the chosen institutions. This result is possibly linked to the privatization of health systems in both contexts. Other authors have perceived a lack of implementation of the transparency regulatory framework in both Chile [25,27] and Colombia, which is characterized by the lack of interaction between the public and private sectors [55] and the existence of information systems related to isolated initiatives on numerous occasions [56]. The above—the unevenness or gap between institutions—is evident in the data shown in Figure 1, where 13% of the institutions studied in Colombia had a high level of implementation in the indicators.

### 5.2. Associations Related to Transparency in Health Areas by Country

In the following section, and through the chi-square test, together with Cramer’s V index, the association between the variables part of the study is considered, according to the countries studied. To typify the intensity of the associations, we followed the recommendations of Betancourt and Caviedes [54], who indicate that as a small effect, any association of 0.10–0.30 on Cramer’s V index; a moderate association when the coefficient is in the range of 0.30–0.50; and a high association when it is in the range of 0.50–1. In addition, to be able to include an association, the objective variable had to register at least five cases, which is the recommended number to be able to execute this type of test.

For the variables considered—the publication of the titleholder’s institutional agenda (V4), the publication of data related to the management and quality of health services (V16), and the existence of a button or section, within the website, called “Transparency” or something similar (V23)—we found no significant associations in the four cases studied.

We did not detect three or more significant associations according to the four countries studied in any of the variables addressed. However, in 11 of the variables, we found significant associations between two cases, grouped according to the following blocks to better interpret the results.

#### 5.2.1. Transparency on Economic Indicators

In the publication of the economic remuneration of the holder, there was a moderate association in Spain (χ^2^ (1, N = 64) = 13.136, Cramer’s V = 0.453, *p* < 0.05), although this is higher than that of Ecuador (χ^2^ (1, N = 64) = 9.269, Cramer’s V = 0.381, *p* < 0.05). Similarly, regarding the publication of the salaries of senior positions, a moderate association was found in the case of Ecuador (χ^2^ (1, N = 64) = 13.136, Cramer’s V = 0.453, *p* < 0.05), although it is higher than the association detected in the Spanish case for this variable (χ^2^ (1, N = 64) = 9.269, Cramer’s V = 0.381, *p* < 0.05). The publication of the annual budgets of the institutions was moderately associated in Chile (χ^2^ (1, N = 64) = 12,800, Cramer’s V = 0.447, *p* < 0.05). There was a weak association in Ecuador (χ^2^ (1, N = 64) = 3.951, Cramer’s V = 0.248, *p* < 0.05). Similarly, in both countries, the information on budget execution and annual accounts were linked, although it was moderately linked in Chile (χ^2^ (1, N = 64) = 8.752, Cramer’s V = 0.370, *p* < 0.05) and weakly linked in Ecuador (χ^2^ (1, N = 64) = 4.923, Cramer’s V = 0.277, *p* < 0.05).

#### 5.2.2. Transparency on Personnel and Recruitment

The publication of the jobs of the workforce and civil servants was moderately associated in Ecuador (χ^2^ (1, N = 64) = 9.223, Cramer’s V = 0.383, *p* < 0.05) and weakly associated in Spain (χ^2^ (1, N = 64) = 5.119, Cramer’s V = 0.285, *p* < 0.05). Moreover, in Ecuador, the publication of job offers or public tenders had a moderate effect (χ^2^ (1, N = 64) = 11.175, Cramer’s V = 0.418, *p* < 0.05), although this is higher than in the case of Spain (χ^2^ (1, N = 64) = 7.481, Cramer’s V = 0.342, *p* < 0.05). In the Colombian case, displaying a graphic organization chart on the web with the names or functions of the top managers showed a moderate association (χ^2^ (1, N = 64) = 14,139, Cramer’s V = 0.470, *p* < 0.05), coinciding with the existing association in the Spanish case (χ^2^ (1, N = 64) = 14,139, Cramer’s V = 0.470, *p* < 0.05). For its part, the information on the regulations that develop and apply the basic organic structure had a moderate association in Ecuador (χ^2^ (1, N = 64) = 9.007, Cramer’s V = 0.375, *p* < 0.05) and a weak one in Chile (χ^2^ (1, N = 64) = 4.217, Cramer’s V = 0.257, *p* < 0.05).

#### 5.2.3. Transparency on the Basic Functions Institutions

The information on the functions of the entities/bodies linked to health areas had a moderate association in Spain (χ^2^ (1, N = 64) = 6.172, Cramer’s V = 0.311, *p* < 0.05) and a weak one in the case of Colombia (χ^2^ (1, N = 64) = 4.805, Cramer’s V = 0.274, *p* < 0.05). Furthermore, the publication of service charters was highly associated in Ecuador (χ^2^ (1, N = 63) = 28.169, Cramer’s V = 0.669, *p* < 0.05) and weakly associated in Spain (χ^2^ (1, N = 63) = 4.006, Cramer’s V = 0.252, *p* < 0.05).

#### 5.2.4. Transparency on the Accessibility and Data of Institutions

The possibility of downloading data provided by the institution in reusable formats was associated with a moderate effect in Spain (χ^2^ (1, N = 64) = 8,529, Cramer’s V = 0.365, *p* < 0.05) but descends in the Colombian case until it is characterized as weak (χ^2^ (1, N = 64) = 5.316, Cramer’s V = 0.288, *p* < 0.05).

The previous four groups summarize the coincidences in the associations between the case studies. Nevertheless, 10 associations were detected in only one of the countries, as follows in the Table 2.

Among the main unique associations, the publication of the cost of institutional advertising campaigns in the Ecuadorian media stands out, with a high effect (χ^2^ (1, N = 64) = 40.727, Cramer’s V = 0.798, *p* < 0.05). In Spain, conversely, we find high associations in the assets declaration of the titleholder of the institution (χ^2^ (1, N = 64) = 34.158, Cramer’s V = 0.731, *p* < 0.05) and the publication of the resume of the related senior positions (χ^2^ (1, N = 64) = 17.417, Cramer’s V = 0.522, *p* < 0.05).

## 6. Conclusions

In global terms, we found an irregular concept of transparency in the public administration of the four countries investigated. The two countries with public health infrastructure (Ecuador and Spain), in comparison, have greater measures of transparency than those with mixed public–private health systems do (Chile and Colombia). In those cases, citizens are not conceived of as auditors, [3,12] nor are key aspects such as public income and expenses shown in a profound manner [40]. Instead, we observed that a conceptualization of transparency is imposed on websites associated with digital repositories. That is to say, according to what is recommended by the previous literature, we consider that, largely, citizen participation is restricted [6] and that the possible reinterpretations and appropriations of public information are made by journalists, as described by Schudson [7].

We are aware of the diversity of definitions that guide the concept of transparency [8,9], even more so in the four countries herein, which present different normative frameworks, cultural perspectives, and levels of internet access. Thus, as in comparative studies such as that by Huijboom and van den Broek [16], we have found various conceptions anchored to the chosen public administrations. However, their common denominator, although it has nuances, is the scarce civic commitment of these websites to the citizens that the websites serve and represent. Instead of promoting an audit of the contents [12] or an active search for information and even personalized access [10], the dispersion of the data suggests constant omissions of information and a hyper-concentration motivated by an obligation to comply with a specific law. For example, on this point, the remuneration of the positions and employees is sometimes offered with codes that are not necessarily known and may be unintelligible, in just the same manner as budget execution, if available. In addition, data are not updated continuously; for different items, the data or documents were found dated 2–3 years before the date of observation.

However, apart from a generally irregular implementation of the indicators proposed in the selected sample, something substantively differentiates the four countries examined. Two have mixed public–private health systems, namely, Chile and Colombia; the other two, Ecuador and Spain, have public health systems. As was stated, this seems to be a significant difference between both cases in terms of transparency: in the websites of the institutions located in countries with public systems, we found generally higher levels of implementation of quality indicators. In addition, this conclusion coincides with those of some previous studies, which have highlighted the lack of transparency of Chilean administration [25], the problems of access to information in this country [26], and the gap and the low participation that is encouraged by Colombian institutions [28,29].

In the cases of Chile and Colombia, in general, there is an insufficient effort to offer transparent and quality information. In the Chilean case, the transparency portal (at https://www.portaltransparencia.cl, accessed on 28 April 2021) of the Council for Transparency publishes the information that public administrations must provide in the field of active transparency. However, the portal comprises repositories of documents that necessitate technical knowledge and time to arrive at the information required for consultation; consequently, the use of such documents among citizens is generally not encouraged. Almost all administrations have a link on the homepage of their institutional website that leads to the portal; thus, these administrations, for the most part, seem to consider this sufficient, except for a few websites wherein specific spaces with some information have been provided. However, in most cases, the information found is scattered, out of date, not treated to make it comprehensible, and difficult to consult owing to technical lethargy as is the case of, for example, illegible organizational charts, which are published as poor quality images.

In Colombia, a gap was perceived between some administrations and others. As the institutional scope decreases—that is, in the less populated municipalities—a more confusing management of transparency is observed, one that is more attached to rigorous notions that assimilate the regulatory framework, introduce it as a repository, or directly avoid introducing it. Differences between national and local institutions have already been highlighted in the observation of other contexts [18,19,20]. Thus, in the largest cities or the ministry, there are meritorious attempts to facilitate active transparency and attention to citizens. In this country, therefore, the provision of websites for health facilities is possibly affected by the scarcity of resources—both human and material—which translates into irregular strategic planning of transparency.

By contrast, in the countries with a public health system, namely, Ecuador and Spain, there does not seem to be a clear correlation between the most populated municipalities and better scores. In the Spanish case, the greater availability of information concerning the field of health in autonomous communities is due to the regulation of territorial powers in this country. According to Article 148 of the Spanish Constitution (BOE, no. 311, of 29 December 1978), the autonomous communities have competencies in this area. As established by Law No. 14 of April 25, 1986, General Health, in Article 42, these administrations oversee the organization of their respective health services while accounting for the responsibilities and competencies of the provinces and municipalities. Within the municipalities, the smaller size of these administrative entities entailed that some issues related to health (for example, public employment, budgets, budget execution, and signed contracts, to name a few) appeared jointly with all the other areas of administrative action. There were items on which less information was offered, both in the autonomous communities and in the municipalities of Spain. These were the costs of institutional advertising campaigns in the media, the agenda of those responsible for health, and the functions of the bodies that are part of the basic organic structure in this area, all of which are necessary to monitor the management of these administrations. Moreover, in the case of municipalities, information on health quality and the characteristics of the campaigns is rather scarce.

In Ecuador, regulatory advances have been providing a legislative framework conducive to transparency; however, at the municipal level, the information recorded is not sufficient to conclude that the legal framework has been complied with. It is evident that information is scarce and complies with the minimum requirements for it to be considered complete and detailed as stipulated by the LOTAIP. At the prefectural level, the situation is similar to that of the municipal administration; that is to say, although information is published, it does not reach full compliance on issues related to transparency, which also occurs with the ministry. In this case, the published information fails to fulfill the minimum criteria that must be met for the information to be considered transparent, and a complete precariousness is noted in the publications made by the institution in general.

Some limitations regarding this research study should be noted. Although we have examined four countries, three of which represent two Ibero-American health system trends, if the research technique is applied to other countries, the results may vary depending on each legislative and administrative case. In addition, in future analyses, it would be interesting to expand the sample of regions and municipalities and with it the research technique toward an incorporation of qualitative methods that help deepen the investigation. Although we have considered the larger entities and can therefore assume that the smaller ones will present either similar or worse results, studies that validate or repudiate this possibility must be conducted. Furthermore, the competencies of each administration level in health matters in each country raise the need to design flexible indicators and differentiate national particularities without losing sight of the need to have a procedure that allows for a comparative assessment of transparency in different Ibero-American states. That is to say, beyond the specific contributions presented herein, we believe that one of the strengths of the present study is its proposal of a methodology designed to overcome the barriers of transnational, national, regional, and local comparisons of transparency, which may prove useful for other investigations with a similar orientation.

## Figures and Tables

**Table 1:** Transparency in the websites of the health areas of the ministries, regions, and municipalities of Chile, Colombia, Ecuador, and Spain (2021).

Indicators	Chile			Colombia			Ecuador			Spain			Total *	
	*M*	*R*	*Mu*	*M*	*R*	*Mu*	*M*	*R*	*Mu*	*M*	*R*	*Mu*	*n*	*%*
V1: Is the resume of the titleholder published?	1	0	1	1	5	1	0	4	9	0	4	7	33	52
	100%	0%	10%	100%	100%	10%	0%	80%	90%	0%	80%	70%		
V2: Is the financial remuneration of the titleholder published?	0	0	0	1	1	4	1	4	9	0	5	10	35	55
	0%	0%	0%	100%	20%	40%	100%	80%	90%	0%	100%	100%		
V3: Are the assets declaration of the titleholder published?	0	0	0	0	2	1	0	1	1	0	5	9	19	30
	0%	0%	0%	0%	40%	10%	0%	20%	10%	0%	100%	90%		
V4: Is the institutional agenda of the titleholder published?	0	0	0	0	0	0	0	1	3	0	1	3	8	13
	0%	0%	0%	0%	0%	0%	0%	20%	30%	0%	20%	30%		
V5: Are the resumes of the related senior officials published?	1	2	1	1	2	0	0	0	0	0	4	7	18	28
	100%	40%	10%	100%	40%	0%	0%	0%	0%	0%	80%	70%		
V6: Are senior officials’ contributions published?	0	0	2	1	1	2	1	4	10	0	5	9	35	55
	0%	0%	20%	100%	20%	20%	100%	80%	100%	0%	100%	90%		
V7: Is the work description from the personnel and civil servant published?	0	0	2	1	3	5	1	4	10	0	5	8	39	61
	0%	0%	20%	100%	60%	50%	100%	80%	100%	0%	100%	89%		
V8: Are the job offers or public tenders published?	0	3	6	1	3	9	1	0	4	1	5	9	42	66
	0%	60%	60%	100%	60%	90%	100%	0%	40%	100%	100%	90%		
V9: Is there a graphical organizational chart with the names and functions of the top managers?	0	0	4	1	4	10	0	0	0	0	5	10	34	53
	0%	0%	40%	100%	80%	100%	0%	0%	0%	0%	100%	100%		
V10: Are the regulations that develop and apply the basic organic structure informed?	1	2	5	1	2	8	1	5	10	1	5	4	45	70
	100%	40%	50%	100%	40%	80%	100%	100%	100%	100%	100%	40%		
V11: Are the functions of these entities/bodies informed?	1	3	7	1	4	8	0	4	4	1	3	1	37	58
	100%	60%	70%	100%	80%	80%	0%	80%	40%	100%	60%	10%		
V12: Are annual and multiyear plans and programs informed?	1	4	5	1	4	10	0	5	10	1	5	5	51	80
	100%	80%	50%	100%	80%	100%	0%	100%	100%	100%	100%	50%		
V13: Are the service charters published?	0	0	0	0	0	0	0	5	10	1	4	4	24	38
	0%	0%	0%	0%	0%	0%	0%	100%	100%	100%	80%	44%		
V14: Is the annual budget published?	0	2	7	1	4	10	1	5	10	1	5	8	54	84
	0%	40%	70%	100%	80%	100%	100%	100%	100%	100%	100%	80%		
V15: Is the information on budget execution and annual statements published?	0	2	7	1	4	9	1	5	10	0	5	8	52	81
	0%	40%	70%	100%	80%	90%	100%	100%	100%	0%	100%	80%		
V16: Are data related to the management and quality of health services published?	1	3	4	0	1	1	1	1	5	1	4	1	23	37
	100%	60%	40%	0%	20%	10%	100%	25%	50%	100%	80%	10%		
V17: Are the formalized contracts published?	0	0	4	1	2	0	0	3	8	0	5	9	32	50
	0%	0%	40%	100%	40%	0%	0%	60%	80%	0%	100%	90%		
V18: Are the signed agreements published?	0	0	3	1	0	0	0	4	5	0	5	6	24	38
	0%	0%	30%	100%	0%	0%	0%	80%	50%	0%	100%	67%		
V19: Are the subsidies awarded published?	0	0	4	1	2	5	1	3	10	1	4	7	38	59
	0%	0%	40%	100%	40%	50%	100%	60%	100%	100%	80%	70%		
V20: Is the cost of institutional advertising campaigns published in the media?	0	0	0	1	2	0	1	5	10	0	0	3	22	34
	0%	0%	0%	100%	40%	0%	100%	100%	100%	0%	0%	30%		
V21: Are the characteristics of institutional advertising campaigns published in the media?	1	1	0	0	1	0	0	1	0	1	5	2	12	19
	100%	20%	0%	0%	20%	0%	0%	20%	0%	100%	100%	20%		
V22: Is there a possibility of downloading data provided by the institution in reusable formats?	0	0	2	1	4	5	0	0	0	1	4	5	21	33
	0%	0%	20%	0%	80%	50%	0%	0%	0%	100%	80%	50%		
V23: Is there a button or section, within the website, called “Transparency” or something similar?	1	2	9	1	2	9	1	5	9	1	4	10	54	84
	100%	40%	90%	100%	40%	90%	100%	100%	90%	100%	80%	100%		
V24: Is there some kind of strategy on the website to favor the accessibility of ethnic groups or nationalities?	0	3	1	0	3	2	0	0	0	1	3	4	17	27
	0%	60%	10%	0%	60%	20%	0%	0%	0%	100%	60%	40%		

Source: authors’ elaboration. Note: M = ministry; R = departments, prefecture, or autonomous communities; Mu = municipalities. * *Total* describes both sample frequencies (*n*) and the percentage (*%*) after the sum of all the cases analyzed.

**Table 2:** Unique transparency associations on the websites of the health areas of the ministries, regions, and municipalities of Chile, Colombia, Ecuador, and Spain (2021).

Country	Variable	χ^2^	gl	N	Cramer’s V	Interpretation
Chile	Are annual and multiyear plans and programs informed?	3.893	1	64	0.247	Weak
Ecuador	Is the resume of the titleholder published?	7.528	1	64	0.343	Moderate
Ecuador	Are the subsidies awarded published?	6.996	1	64	0.331	Moderate
Ecuador	Is the cost of institutional advertising campaigns published in the media?	40.727	1	64	0.798	High
Spain	Is the assets declaration of the titleholder published?	34.158	1	64	0.731	High
Spain	Are the resumes of the related senior officials published?	17.417	1	64	0.522	High
Spain	Are the formalized contracts published?	12.000	1	64	0.433	Moderate
Spain	Are the signed agreements published?	10.366	1	64	0.406	Moderate
Spain	Are the characteristics of institutional advertising campaigns published in the media?	13.675	1	64	0.462	Moderate
Spain	Is there some kind of strategy on the website to favor the accessibility of ethnic groups or nationalities?	6.008	1	64	0.306	Moderate

Source: authors’ elaboration. Note: all the associations are significant (*p* < 0.05).

## Data Availability

No restrictions apply to the availability of these data. All the data were captured from the public websites of the institutions indicated in the methodology section.
